# Artificial intelligence automates the characterization of reversibly actuating planar-flow-casted NiTi shape memory alloy foil

**DOI:** 10.1371/journal.pone.0275485

**Published:** 2022-10-19

**Authors:** Ritaban Dutta, Ling Chen, David Renshaw, Daniel Liang

**Affiliations:** 1 CSIRO DATA61, Hobart, Australia; 2 CSIRO Manufacturing, Clayton, Australia; Faculty of Engineering, University of Rijeka, CROATIA

## Abstract

Nickel-Titanium (NiTi) shape memory alloys (SMAs) are smart materials able to recover their original shape under thermal stimulus. Near-net-shape NiTi SMA foils of 2 meters in length and width of 30 mm have been successfully produced by a planar flow casting facility at CSIRO, opening possibilities of wider applications of SMA foils. The study also focuses on establishing a fully automated experimental system for the characterisation of their reversible actuation, significantly improving SMA foils adaptation into real applications. Artificial Intelligence involving Computer Vision and Machine Learning based methods were successfully employed in the development of the automation SMA characterization process. The study finds that an Extreme Gradient Boosting (XGBoost) Regression model based predictive system experimented with over 175,000 video samples could achieve 99% overall prediction accuracy. Generalisation capability of the proposed system makes a significant contribution towards the efficient optimisation of the material design to produce high quality 30 mm SMA foils.

## Introduction

These shape-memory special behaviours of NiTi SMAs are due to the martensitic-to-austenitic transformation and its reversion, which can be activated by increasing and decreasing its temperature, respectively. NiTi SMAs, mainly in wire, rod and plate forms, have demonstrated its actuation ability as well as other attributes, such as a high corrosion resistance, wear resistance, specific electric resistance, good fatigue properties, and biocompatibility [1]. However, these forms have been tested in a limited number of industrial applications, that neither have restraints on size and volume, nor require a fast, reversible and precision actuation. In all the SMA applications being explored in various areas, e.g., space technology [2], automotive [3], sensing and actuating [4], and biomedical devices [5, 6], the use of Ni-Ti SMAs in miniaturised sensors and actuators attracts great interests. Building a microsystem with the integration of moving mechanisms, sensors and electronics, demands further miniaturization of sensing and actuating devices [7].

Thin SMA foils have many advantages over the SMA wire, rod or plate, namely, the ability to generate a large actuation force, respond faster to thermal stimulus, compact, and low cost, which could be used in the field of miniaturised actuators and thermo-sensor devices. A NiTi SMA component of a small dimension fabricated from the SMA foil can react fast with a predetermined response, when encountering a temperature change, making it ideal as a micro-size device integrating sensing and actuating functions in a microsystem [8].

However, manufacturing NiTi foils is difficult via the existing ingot casting and rolling route, as a result of high reactivity and poor formability of the NiTi alloy. One fabrication technology under development is so-called planar flow casting, which solidifies molten metal into solid foils directly [9]. As the intermediate processing steps are eliminated, the planar-flow-cast NiTi SMA foils are low-cost and can be produced in large volumes.

The planar flow casting is a rapid solidification process that quenches molten metal into solid foils at a cooling rate in a range of 103 to 107 K/s. This rapid solidification can form additional metastable phase [10, 11], which would affect the actuating behaviours of the NiTi SMA foils. The actuating characteristics of the planar-flow-casted NiTi SMA foil was investigated in terms of phase transformation temperature, shape memory effects in terms of actuation force, response time, and degree of actuation [12–14].

**Fig 1:**
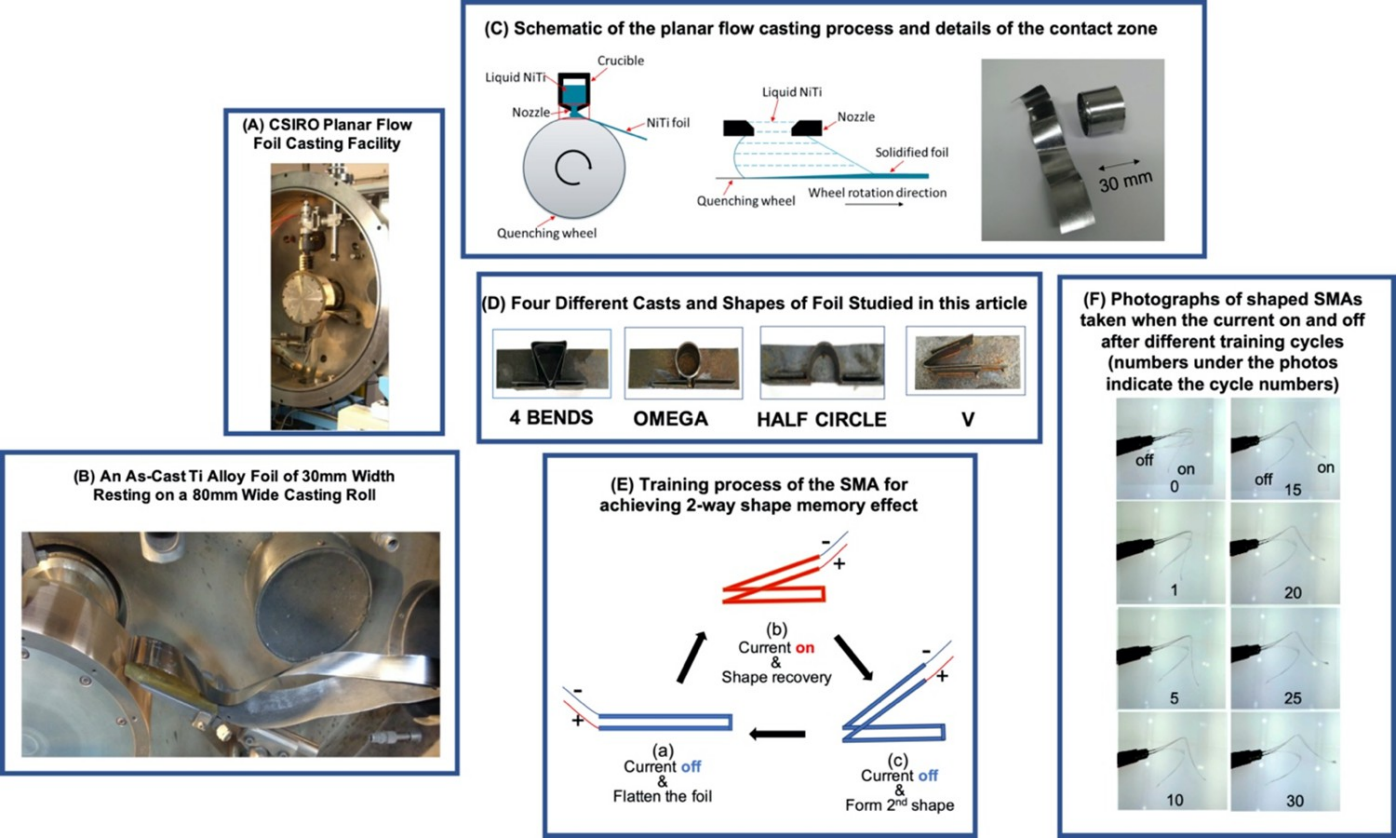
Near-net-shape NiTi SMA foils of long length and larger width of 30 mm have been successfully produced by a planar flow casting facility at CSIRO. (A) CSIRO planar flow foil casting facility, (B) An as-cast NiTi alloy foil of 30 mm width resting on an 80 mm wide casting roll, (C) Schematic of the planar flow casting process and details of the contact zone, (D) Moulds of our different shapes of foils studied in this project, (E) Training process of the SMA for achieving 2-way shape memory effect, and (F) Photographs of shaped SMAs taken when the current is ON and OFF after different number of training cycles.

Near-net-shape NiTi SMA foils of about 50 μm thickness, 2 meters of length and width of 30 mm have been successfully produced by a planar flow casting facility at CSIRO. Foil with a width of 30 mm can be formed into various shapes of SMA components, enabling NiTi SMA to be utilized in a wider range of applications. This advancement at CSIRO Australia creates scientific opportunities to envisage the advantage of the unique behaviour of SMAs (see Fig 1(A)–1(C)).

With the unique advancement of the capability to produce the 30 mm wide SMA foil, there was a need to develop an automated system to rapidly characterize the performance of these foils, enabling a pathway to optimize SMA materials and high-quality control of the manufacturing process. The foils of 30 mm width could be shaped into many geometries and sizes to accommodate a wide range of applications with requirements of various degrees of freedom. The evaluation and quantification of the performance of a SMA foil is often determined by the achieved actuation angles and the amount of force it can generate during thermally excited actuation and recovery transitions. Hence, a system that can accurately and rapidly predict force and angle being generated by an actuating SMA foil under excitement, has evidently become essential [15–20]. Elimination of time-consuming physical testing was a major factor to increase the efficiency of the SMA material characterization and new SMA material discovery [21–26].

A system capable of predicting the potential achievable actuation angles and the amount of force that in the past can be only generated by many experiments on the SMA foil, could make the SMA material characterization significantly faster, hence also making the material design more efficient and optimised. We aimed to design an automated and rapid SMA characterization system to achieve two primary objectives [27–33]. Firstly, we established a capability (aim A) to predict possible actuation angles achievable by a freshly manufactured foil, purely based on the dynamic maximum body temperature of the foil while under the thermal excitement. Secondly, we developed a unique capability (aim B) to predict a possible amount of force that could be generated by that foil while actuating and recovering under the thermal excitement.

In this study, a new methodology was developed, which employed infrared video imaging of the foils under excitements, conventional Computer Vision based video data analysis techniques and Machine Learning based predictive techniques to rapidly characterize the planar-flow-cast NiTi SMA foils [14–19]. After a comparative study using various ensemble machine learning techniques, we employed Extreme Gradient Boosting (XGBoost) [20–27] Regression model in the proposed methodology as the most effective predictor. A large database was generated using in-house automated testing facilities at CSIRO, Australia. The methodology was tested on a very large number of SMA foil samples to establish its overall performance accuracy and generalisation capability while predicting actuation angles and force. The methodology was also adapted as a stand-alone predictive system to predict potential force from unknown foil samples. The system was tested using a blind validation procedure. The blind validation was highly successful showcasing the high potential and effectiveness of the new methodology in the field of SMA foil-based applications [34–38].

## Methods

### Initial shape setting by heat treatment

SMA strips of 30 mm width and 120 mm length were firstly cut out from planar-flow-cast foils. Then they were inserted into the slot of steel moulds with four different shape fixtures, namely, “4 BENDS”, “HALF CIRCLE”, “OMEGA” and “V” (as shown in Fig 1(D)). The assemblies of the moulds and foils were placed inside a tube furnace, undergoing annealing heat treatment to set the shape. The heat treatment was conducted at 550°C, 650°C and 750°C, respectively, for 30 minutes under argon before quenching in water. After removal from the mould, a centre slot with a width of 4 mm and a length of 115 mm was cut out from the strip to enable an electrical current path. before creating four groups of foils according to four different selected shapes.

### Phase transformation temperatures

Austenite start temperature (A_s_) is the temperature at which the SMAs start to actuate when the martensitic-to-austenitic phase transformation begins. Austenite finish temperature (A_f_) is the temperature when the phase transformation is finished. Similarly, when the temperature decreases, the phase transformation from austenite to martensite phase begins at martensite start temperature (M_s_) and completes at martensite finish temperature (M_f_). The phase transformation temperatures were measured by Differential Scanning Calorimetry (DSC, Mettler Toledo DSC3). M_s_, M_f_, A_s_, and A_f_ of planar-flow-cast SMA foil samples annealed at different temperatures were extrapolated through the tangential line method from the DSC measurements and are summarised in Table 1. The results in Table 1 showed that the transformation temperatures decreased with increasing heat treatment temperature. This result suggests that, instead of varying the chemistry, the heat treatment can change the phase transition temperature and thus the actuation temperature. This observation indicates a possible low-cost control over the performance of the NiTi SMA foils.

**Table 1:** The heat treatment of the SMA foils was conducted at 550°C, 650°C and 750°C, respectively, for 30 minutes under argon before quenching in water. The transformation temperatures Ms, Mf, As, Af of both planar-flow-cast SMA foil samples annealed at different temperatures.

Material	Annealing temperature (°C)	M_s_ (°C)	M_f_ (°C)	A_s_ (°C)	A_f_ (°C)
Planar-flow-casted SMA Foils	No heat treatment	60.85	42.72	73.09	94.02
	550	60.8	42.33	72.22	89.54
	650	11.92	-2.57	21.33	42.79
	750	4.97	-3.41	19.69	37.12

### Training of two-way shape memory alloy foils

All the planar-flow-cast NiTi-based strip SMA samples in the sequent training tests were annealed at 550°C. A thermomechanical treatment, known as a training process, was carried out on the SMA foils, enabling them to remember two shapes i.e., two-way actuation. An example of the training process with a foil of “V” shape is shown in Fig 1(E): (a) Deform the sample into the flat strip; (b) Heat up the flat strip by applying a 5A (2.5V) current. The strip recovers to the “V” shape; (c) Turn off the current and wait until the strip cools down, forming the second shape; (d) Repeat (a) to (c) steps for 30 times.

### Effect of training cycle on actuation

After the 1^st^ training cycle, the SMA sample started to show the 2-way shape memory behaviour–i.e., the SMA strip moved to the actuated position when the electrical current was on and shifted back to another position when the current was off. As shown in Fig 1(F), their actuation behaviour during the training cycle with the current on (stimulated) or off (un-stimulated) was photographed after 0, 1, 5, 10, 15, 20, 25 and 30 training cycles of the SMA actuation taken. After the 1^st^ training, the current-on angle is 64° and the current-off angle is 49° with 15° difference. With more subsequent training, the angle difference increased to 30° and the current-on and current-off angles were stabilized at 134° and 102°, respectively, by 30^th^ cycle, with a maximum angle difference at 32°. Each of the foils used in this study went through a consistent 30 cycles of training. A comprehensive summary of the maximum and mean actuation angles achieved by the four different shaped SMA foils have been provided in Table 2.

**Table 2:** A comprehensive summary of the maximum and mean actuation angles achieved by the four different shaped SMA foils, whereas BC is the Body Centroid of the SMA foil, and the Tip is the actuation angle at the dynamically changing tip of the body.

Planar-flow-casted SMA Foil	Max Actuation Angle at Tip	Mean Actuation Angle at Tip	Max Actuation Angle at BC	Mean Actuation Angle at BC
4 BENDS	48°	34°	60°	39°
OMEGA	55°	47°	48°	33°
HALF CIRCLE	58°	45°	54°	37°
V	55°	42°	53°	45°

### Microstructure analysis

**Fig 2:**
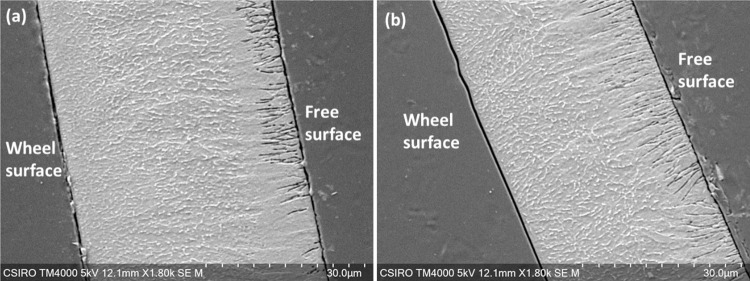
SEM micrograph of the cross section of the etched (a) as cast and (b) 550°C heat treated NiTi foil.

The characterization of the shape memory behavior is highly correlated to phase transformation temperatures, training and actuation. However, one of the most critical aspects of the shape memory training is the optimization of the microstructure. We conducted microstructure analysis on the SMA material used in this research study (Fig 2). The cross-sectional microstructure of the as-spun and the heat-treated foils were examined by a scanning electron microscope (SEM, Hitachi TM4000Plus) after etching with ASTM 151 solution of HF: HNO3: H2O = 6:15:90 (in volume).

### Measurement of generated force

**Fig 3:**
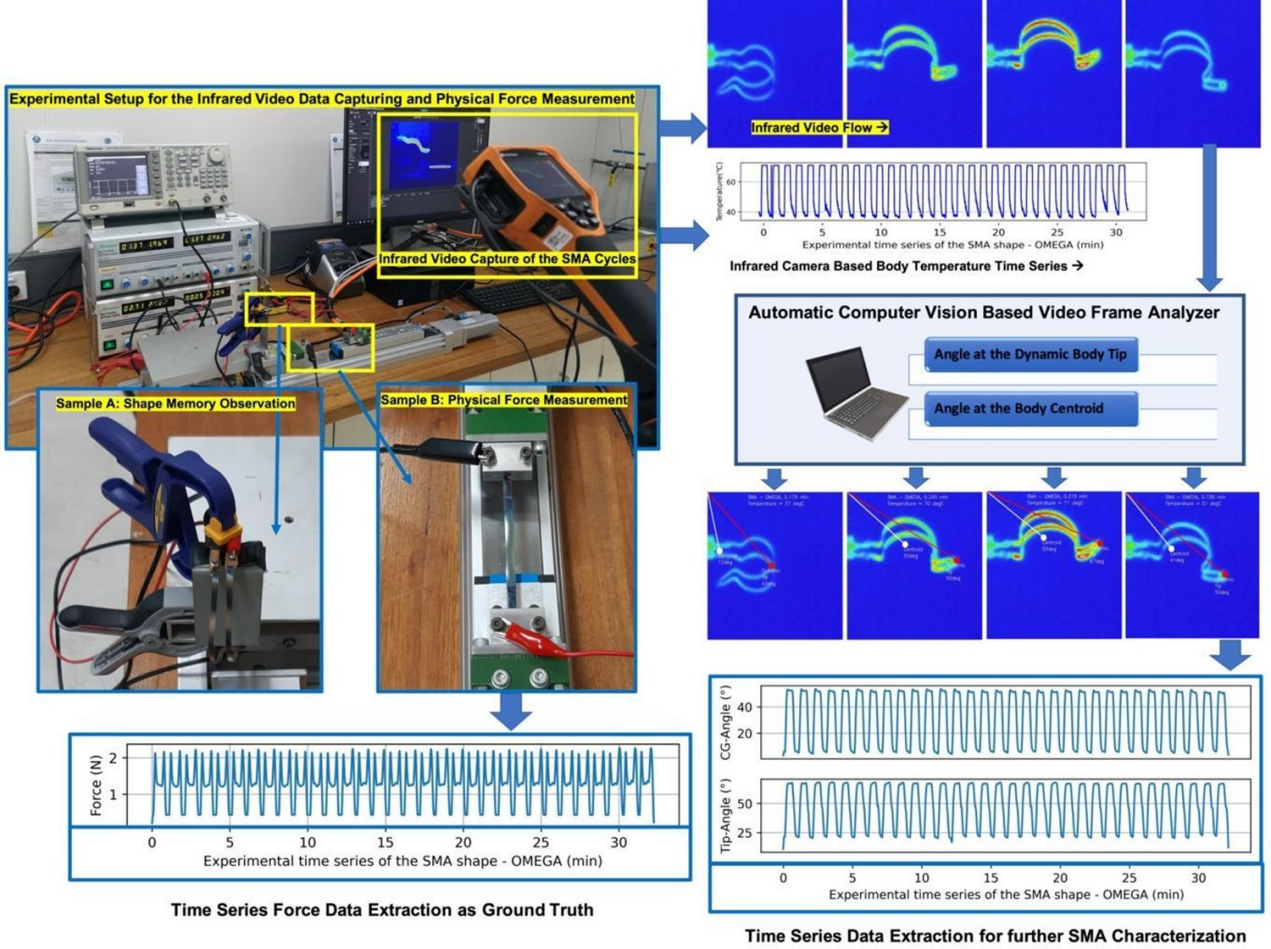
Near-net-shape NiTi SMA overall automated experimental setup consists of two test rigs with automated data logger, an infrared camera with automated data logger, a computer vision system for dynamically analysing video stream, extracting relevant time series data and selecting significant video frames, to be analysed and utilised to train and develop a predictive system. Sample A of the foil was used for infrared video imaging and digitally measuring actuation angles, while the Sample B was used to measure the generated force. This was done simultaneously, to capture a large database for further system implementation.

**Fig 4:**
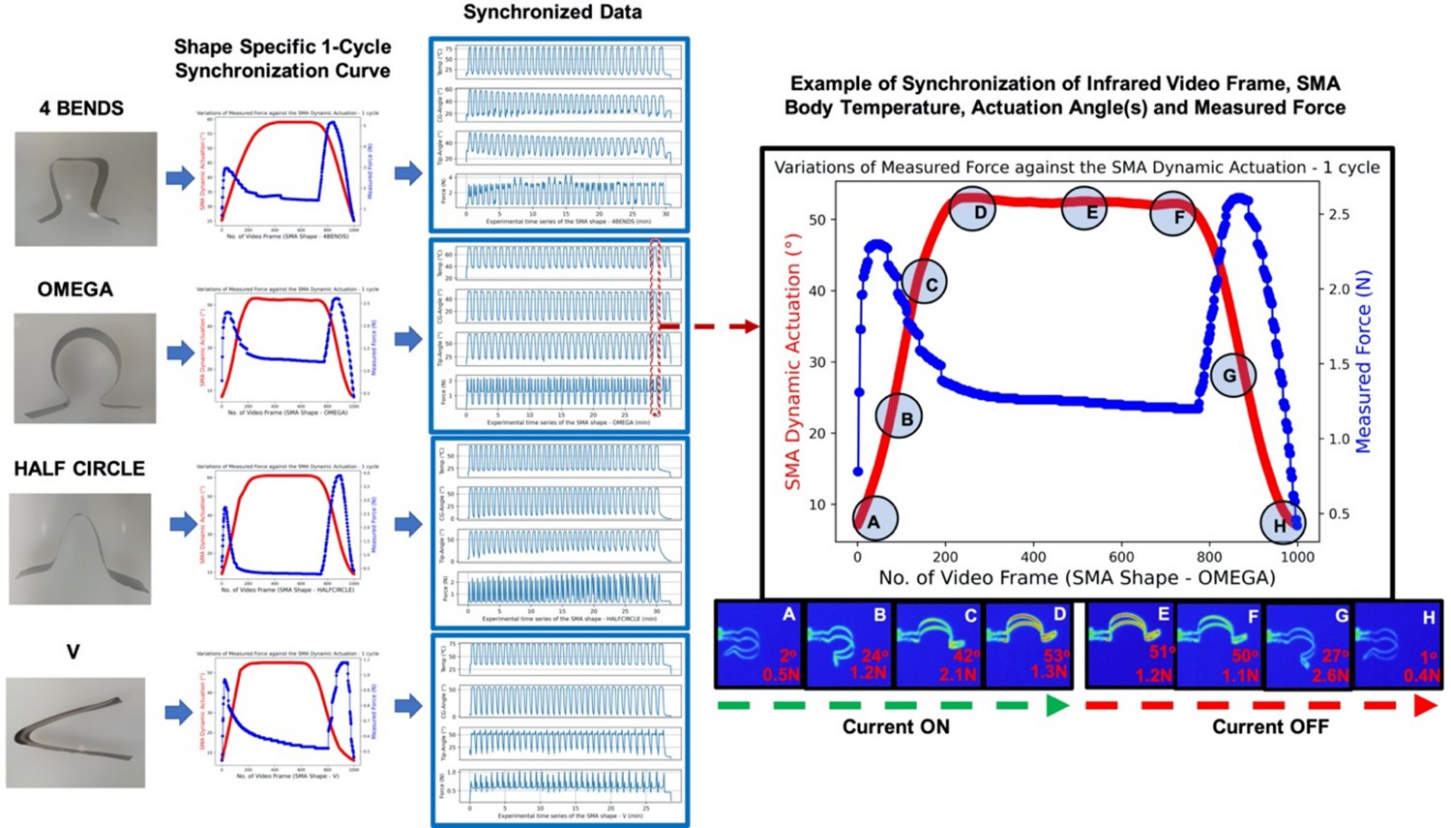
This figure shows the data synchronization procedure. The force, angles, temperature time series and selected video frames were synchronized against time stamps. It also shows an example of utilization of the 1-cycle synchronization curve for a larger data synchronization.

Force generated by the stimulated SMA foils during actuation and recovery were measured as ground truth data. The tests were carried out using an inhouse morphing tester equipped with a 20 Kg load cell. The testing system is shown in Fig 3 while Fig 4 shows the data synchronization procedure. Both ends of SMA sample were loaded to the tester by screws. Torque wrench was used to tighten the screws, ensuring the same clamping force is applied to both ends of the samples. Using a DC power supply (DPD3030, Manson), 7A current was applied to the SMA foil. The generated force was detected by the load cell as the two end sides of the SMA were restricted.

The maximum forces measured during actuation (current on) and recovery (current off), and the corresponding generated force calculated are tabulated in Table 3. The peak force during actuation for the planar-flow-cast material is 4.2 N and for the recovery stage is 2.7 N, while experimenting with the shape called “4 BENDS”. The times needed for the four different shaped SMA bodies to actuate (TA) and recover (TR), are presented in Table 3. “V” shaped sample showed shortest actuation and recovery times, suggesting the fastest response.

**Table 3:** The maximum forces measured during actuation (current on) and recovery (current off) and the times needed for the four different shaped SMA bodies.

Planar-flow-casted SMA Foil	Max Actuation Force (N)	Actuation Time (s)—TA	Max Recovery Force (N)	Recovery Time (s)—TR
4 BENDS	2.7	3	4.2	8
OMEGA	2.1	4	2.27	9
HALF CIRCLE	2.3	2	2.73	8
V	0.7	3	1.02	7

### XGBoost regression model

XGBoost stands for Extreme Gradient Boosting. The XGBoost regression adapts and implements the gradient boosting decision tree algorithm. It implements machine learning algorithms under the Gradient Boosting framework. XGBoost provides a parallel tree boosting (also known as GBDT, GBM) that solves many data science problems in a fast and accurate way [14–20]. In the Gradient boosting approach, in each step of the learning, new models are created that predict the residuals or errors of the prior models. The predictions from the new models are then added together to make the final prediction. XGBoost is an implementation of gradient boosted decision trees designed for speed and performance.

In this study, the important parameters for an instance of the best performing XGBoost regressor model, were set as, booster = ’gbtree’, importance_type = ’gain’, learning_rate = 0.1, n_estimators = 350, max_depth = 8, gamma = 0.5, random_state = 42, subsample = 0.6, min_child_weight = 2.1, max_delta_step = 4, sampling_method = ‘uniform’, {’colsample_bytree’:0.5, ’colsample_bylevel’:0.5, ’colsample_bynode’:0.5}, lambda = 0.9, alpha = 0.2, tree_method = ‘auto’, scale_pos_weight = 1, updater = ‘prune’, refresh_leaf = 0, and grow_policy = ‘depthwise’.

### Predictive experimental design

We tested a capability (aim A) to predict possible actuation angles achievable by a given foil, purely based on the dynamic maximum body temperature of the foil while under the thermal excitement. We utilised a multi-output mode of the XGBoost regression model to predict both actuation angles simultaneously with the single temperature input.

**Fig 5:**
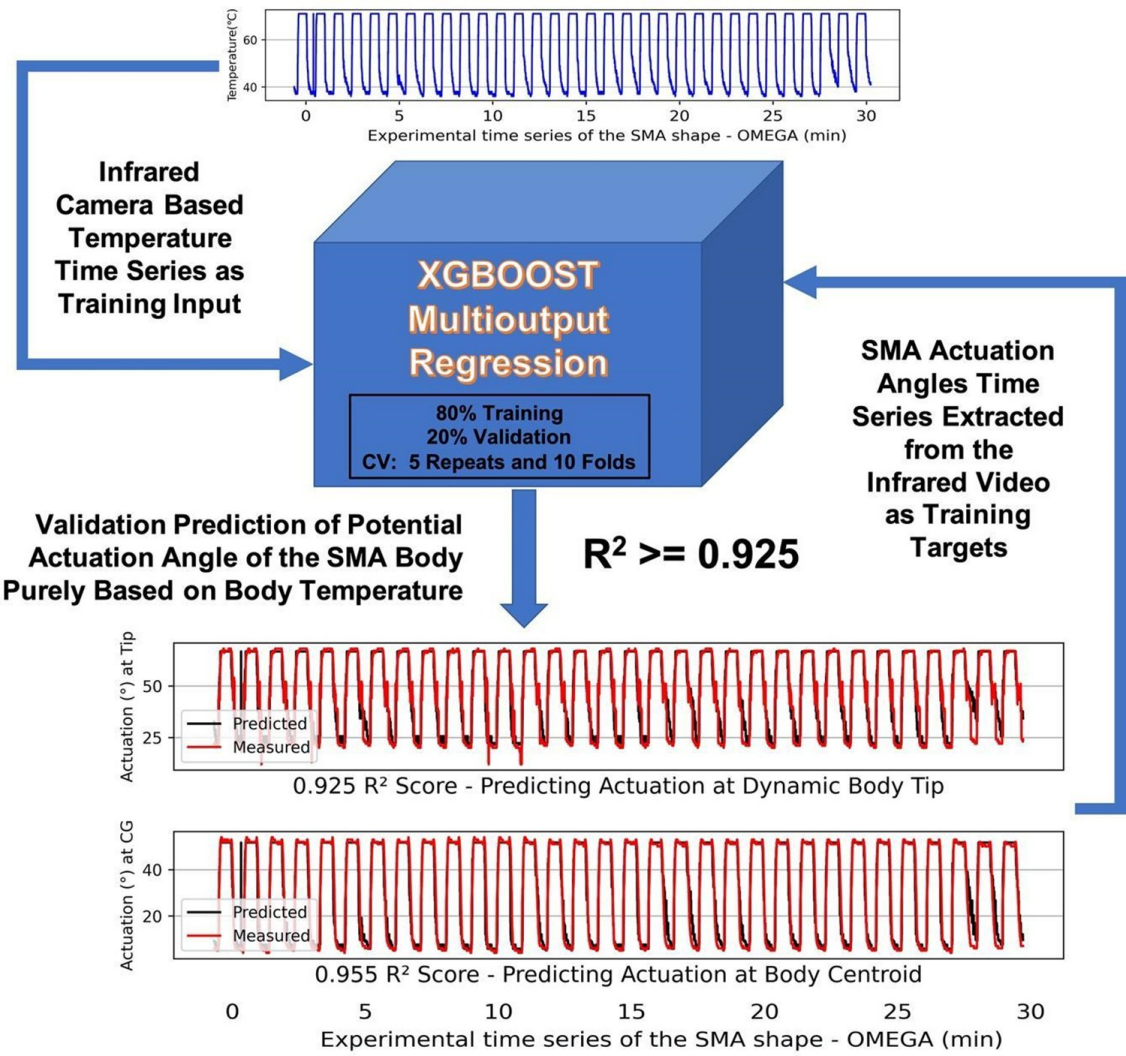
Results of prediction for the aim A. A XGBoost regression model was the best model to predict possible actuation angles (at the BC and at the Tip) with a R^2^ score in a range of 0.925–0.974, while taking the dynamic maximum body temperature as the only input to the model, achieving a mean accuracy of 95%. An example of prediction results is depicted in the figure using an Omega shaped SMA.

Training input for this modelling was the dynamic maximum body temperature of the foil, while the learning targets were the actuation angles. The XGBoost model was able to establish the correlation between temperature and actuation angles with high accuracy (as shown in Fig 5).

Secondly, utilizing the same XGBoost Regression model (separately trained), we developed a unique capability (aim B) to predict a possible amount of force that could be generated by a given foil under the thermal excitement. Training input for this modelling was the video data, while the learning target was the measured force.

**Fig 6:**
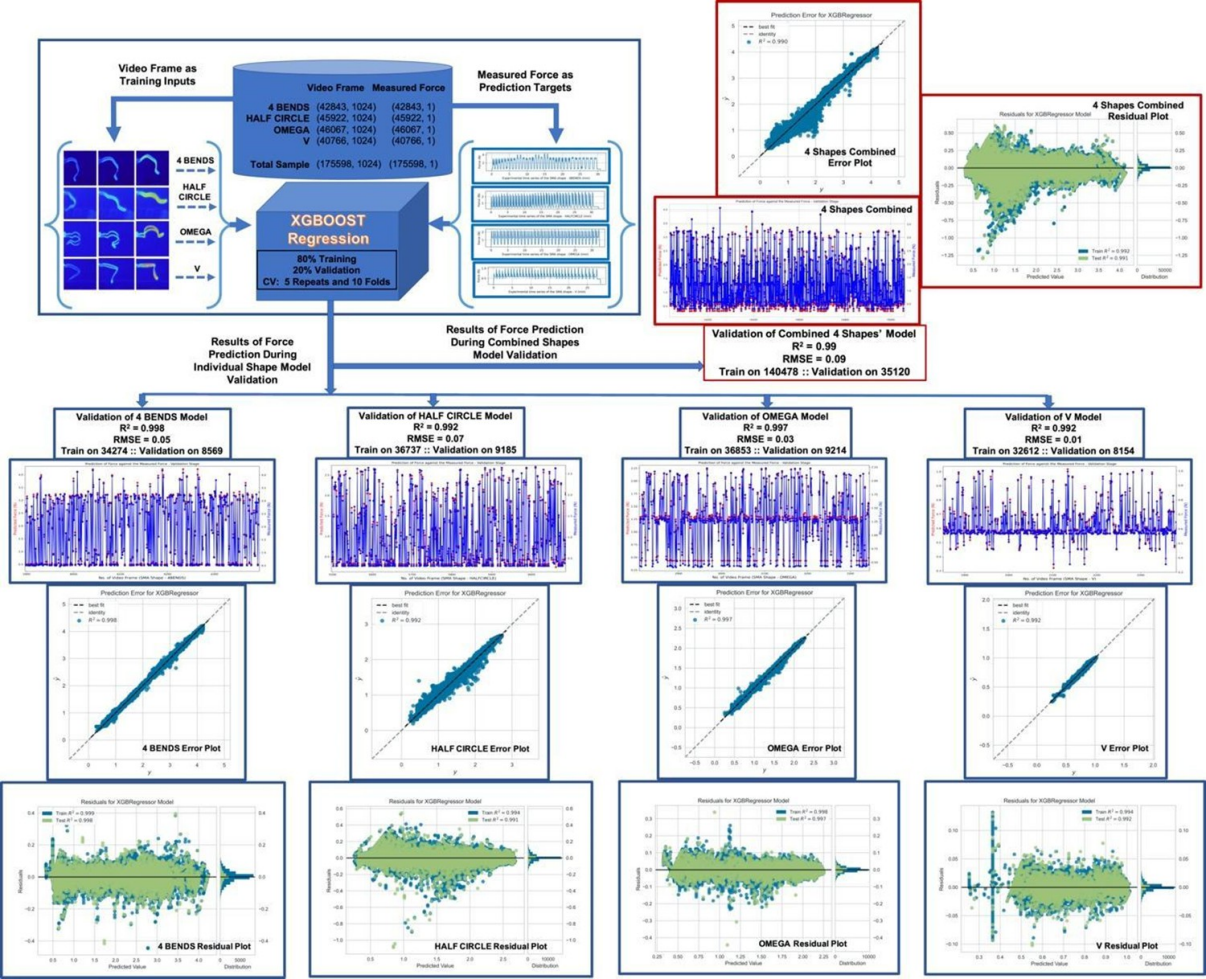
Results of prediction for the aim B. A XGBoost regression model was the best model to predict possible amounts of force with a R^2^ score in a range of 0.99–0.998, achieving a mean accuracy of 99.5%. This figure shows the schematic diagram of the infrared video data-based force prediction capability, input and outputs of the model, and the prediction results consists of mean R^2^ score, Root Mean Square Error (RMSE), Error plots and Residual plots.

This part of the predictive system was trained on the compressed and flattened pixels from each of the selected video frames. All together five different scenarios were experimented, individually, a data set related to each of the four shaped SMA foils and combining four of them as a single data set (as shown in Fig 6).

### The model evaluation

In both parts of predictive modelling, the XGBoost model was trained with 80% of the data (140,478 samples) and tested and validated on the remaining 20% (35,120 samples). A random cross validation (CV) paradigm consists of 5 repeats and 10-Folds (Repeated Folds CV) was used to test the generalisation capability of the model while predicting. We also conducted a separate blind validation of the overall predictive system using unknown foil samples. This blind validation was separate from the Repeated Folds CV during the initial training-testing paradigm of the model development.

Prediction performance for each of the five scenarios have been consistently depicted using, mean R^2^ score, Root Mean Square Error (RMSE), Error plot and Residual plot. In all the Error plots in Fig 6, most of the points are on a straight line at 45° indicating the predictions exactly match the actual measured force data. In the Residual plots there is no significant visible pattern between the predictions and residuals. This showcases that the residuals are uncorrelated and independent. In the distribution part of the residual plots, it is verified that the residuals are approximately normally distributed, highlighting the robustness of the XGBoost model.

## Results

### Fabrication of NiTi SMA foils

A mixture of Ti and Ni metals with Ni/Ti weight ratio of 54.7/45.3 was melted and then cast into foils by a planar flow caster at CSIRO (Fig 1(A)). The planar-flow-cast NiTi SMA foils of about 50 μm thickness and 30 mm width are shown in Fig 1(B). Fig 1(C) shows the schematic of the planar flow casting process and details of the contact zone to produce a 30 mm wide NiTi SMA Foil. The chemical analysis of the foil was performed using an inductively coupled plasma optical emission spectroscopy Varian 730-ES ICP-OES. The results of the analysis showed that the planar-flow-cast SMA foil consisted 49.1% of Ni and 50.9% of Ti.

### Microstructure of the NiTi SMA foils

Fig 2 shows the microstructure of the as-cast and heat-treated NiTi foils with a thickness of about 50 μm. It indicates that the planar-flow-cast foil have coarse-grains at the free surface and fine-grains at the wheel surface. While the grains close to the wheel exhibit equiaxed feature, the grains close to the free surface are columnar feature. These features are due to the cooling rate at the wheel surface are much larger than that at free surface and the directional heat flux in the longitudinal direction of the columnar grains [36, 37]. The non-uniform grain size of the foil could possibly affect the shape memory effect, which are also suggested by other research work [38]. After heat treatment at 550°C, the heat-treated foil shows a similar microstructure with fine and coarse grains at the free surface and the wheel surface regions, respectively.

### Image-based data assimilation

An overview of the system for the automated experimentation is shown in Fig 2. It had two test rigs to excite and test two SMA foil samples (namely Sample A and Sample B in two insets of Fig 2) simultaneously. Each of the SMA foil was tested through 30 repeated cycles, where each cycle consisted of one actuation phase and one recovery phase. Sample A was used for possible actuation under thermal excitement, whereas Sample B was used to measure the force generated by the actuating SMA foil. An infrared camera was used to capture the video data of the actuating SMA foil (Inset box of Sample A in Fig 2), and to extract the dynamic body temperature of the SMA foils, as time series data during actuation and recovery. Body temperature data varied over a range of ~29°C to 79°C and were stored by the infrared camera to the central data server. The captured infrared video data were automatically processed using a live CV based processing system to extract achieved actuation angles, one actuation angle at the Body Centroid (BC) of the SMA foil and the actuation angle at the dynamically changing tip of body (Tip), the maximum stretched tip of the body on a video frame at any given time. The actuation angles were extracted as time series data as demonstrated in Fig 2.

### Image-based measurement of actuation angles

During the processing of the infrared video image processing, each of the video frames were compressed into [32 X 32] pixel frames. The frames were converted into grayscale, eroded and dilated to make the actuating SMA foil only visible in a video frame. This step eliminated any background lighting noise that was recorded during large scale video capturing and simplified the pixel-based feature extraction. Dynamically the X and Y coordinates of the BC and the Tip were calculated from each of the video frames. The top left point of the video frame was considered as the origin, whereas the angle between the origin and the BC, and the angle between the origin and the Tip were calculated and stored as time series (Fig 3).

### Selection of significant video frames

The quality of the captured video was kept consistent during all experimentation in those 239,070 video frames collected from the four groups of differently shaped actuating SMA bodies. Standardizing the experimental protocol was crucial to capture data of a high quality that is consistent and completely reproducible. Selection of video frames to be included in the final study was determined by a significance tolerance factor, defined by the change in actuation angles at BC and Tip, both being greater than 1.5°, an effective minimum angular threshold used in this study. This was to eliminate the repetitive video frames (without any significant changes in achieved actuation) from the overall analysis and any potential bias that could be created by this type of repetition. Finally, 175,598 video samples were selected, and all the video frames were individually flattened into a pixel matrix of size of [1 X 1024], to be used in the predictive analysis and the evaluation of the system.

### Automated synchronization of data

The data synchronization among the force, angles, temperature and selected video frames was critical for the predictive analytics and system development. Initially, one cycle of excited actuation and recovery was recorded for each of the shapes. This one cycle curve was used to benchmark all the subsequent repeat cycles in the process of data synchronization against time. Fig 4 shows shape specific synchronization curves for the four different shaped SMA foils, synchronized time series data mapped together, and a demonstrative example of representative video frames associated with different stages of an experimental cycle (marked as A, B, C, D, E, F, G and H, labelled with the measured force and actuation angle at the Tip, that moment in time) while using OMEGA shaped SMA foil.

Synchronized data set had 175,598 video samples along with the corresponding four recorded attributes, ‘generated force’, ‘actuation angle at BC’, ‘actuation angle at Tip’, and ‘maximum body temperature’. The “4 BENDS” shaped foils were represented with 42843 samples, “HALF CIRCLE” shaped foils were represented with 45922 samples, “OMEGA” shaped foils were represented with 46067 samples, and “V” shaped foils were represented with 40766 samples. All data and examples of processed video files related to this manuscript has been made available in the supplementary section.

### Model prediction accuracy

The study finds an Extreme Gradient Boosting (XGBoost) Regression model was the best model to predict possible actuation angles (at the BC and at the Tip) with a R^2^ score in a range of 0.925–0.974, while taking the dynamic maximum body temperature as the only input to the model, achieving a mean accuracy of 95%. This result successfully benchmarked the (aim A). Fig 5 shows the schematic diagram of the actuation angles prediction capability, input and outputs of the model, and a comparative example of predicted angles against the measured angles, using an OMEGA shaped SMA foil. Table 4(A)–4(E) summarises all mean R^2^ score results from the comparative predictive regression analysis and quantitative selection of the XGBoost Regression model as the best predictor.

**Table 4:** (A) The mean R^2^ score results from the comparative predictive regression analysis while experimenting with “4 BENDS” SMA shapes. **(B)** The mean R^2^ score results from the comparative predictive regression analysis while experimenting with “HALF CIRCLE” SMA shapes. **(C)** The mean R^2^ score results from the comparative predictive regression analysis while experimenting with “OMEGA” SMA shapes. **(D)** The mean R^2^ score results from the comparative predictive regression analysis while experimenting with “V” SMA shapes. **(E)** The mean R^2^ score results from the comparative predictive regression analysis while experimenting with all four SMA shapes combined in a single data set.

**(A)**			
4 BENDS	Temperature Based Prediction (aim A)		Infrared Video Based Prediction (aim B)
Regression Model	R^2^: 4 Bends Tip Angle	R^2^: 4 Bends BC Angle	R^2^: 4 Bends Force
Ada Boost	0.756	0.781	0.892
Extra Tree	0.865	0.845	0.902
Gradient Boosting	0.823	0.844	0.92
XG Boost	**0.926**	**0.974**	**0.998**
Random Forest	0.852	0.876	0.901
**(B)**			

HALF CIRCLE	Temperature Based Prediction (aim A)		Infrared Video Based Prediction (aim B)
Regression Model	R^2^: Half Circle -	R^2^: Half Circle -	R^2^: Half Circle—Force
	Tip Angle	BC Angle	
Ada Boost	0.815	0.823	0.845
Extra Tree	0.876	0.899	0.91
Gradient Boosting	0.912	0.897	0.93
XG Boost	**0.928**	**0.935**	**0.992**
Random Forest	0.892	0.872	0.835
**(C)**			

OMEGA	Temperature Based Prediction (aim A)		Infrared Video Based Prediction (aim B)
Regression Model	R^2^: Omega Tip Angle	R^2^: Omega BC Angle	R^2^: Omega Force
Ada Boost	0.814	0.822	0.831
Extra Tree	0.901	0.912	0.92
Gradient Boosting	0.856	0.873	0.861
XG Boost	**0.925**	**0.955**	**0.997**
Random Forest	0.873	0.881	0.807
**(D)**			

V	Temperature Based Prediction (aim A)		Infrared Video Based Prediction (aim B)
Regression Model	R^2^: V Tip Angle	R^2^: V BC Angle	R^2^: V Force
Ada Boost	0.831	0.805	0.793
Extra Tree	0.875	0.826	0.891
Gradient Boosting	0.912	0.907	0.91
XG Boost	**0.934**	**0.965**	**0.992**
Random Forest	0.917	0.91	0.821
**(E)**			

	Temperature Based Prediction (aim A)		Infrared Video Based Prediction (aim B)
Regression Model	R^2^: Combined	R^2^: Combined	R^2^: Combined Force
	Tip Angle	BC Angle	
Ada Boost	0.789	0.819	0.813
Extra Tree	0.879	0.892	0.91
Gradient Boosting	0.915	0.921	0.856
XG Boost	**0.926**	**0.971**	**0.99**
Random Forest	0.91	0.921	0.872

In addition to the high accuracy of predicting actuation angles, the XGBoost Regression model was also the best model to predict possible amounts of force with a R^2^ score in a range of 0.99–0.998, achieving a mean accuracy of 99.5%. This result successfully benchmarked the (aim B). In Table 5, we summarised some traditional statistical measures including standard deviation (std) and mean of all learning targets, the measured force time series. It was found that among the five-time series, mean varied on a range of 0.64–1.97 and std varied on a range 0.12–1.17. The RMSE varied on a range of 0.01–0.09, indicating a very high accuracy for the selected XGBoost regression model while predicting force purely based on infrared video of the actuating SMA foils under thermal stimulus. Fig 6 shows the schematic diagram of the infrared video data-based force prediction capability, input and outputs of the model, and the prediction results consists of mean R^2^ score, Root Mean Square Error (RMSE), Error plots and Residual plots.

**Table 5:** Some traditional statistical measures from the learning targets, the measured force time series. This was analysed to verify and justify the robustness of the prediction outcomes from a XGBOOST regression model.

	4BENDS	HALFCIRCLE	OMEGA	V	Combined
Prediction RMSE:	0.05	0.07	0.03	0.01	0.09
Prediction R^2^:	0.998	0.992	0.997	0.992	0.99
Count	42843	45922	46067	40766	175598
mean	1.969224	0.997715	1.271821	0.636530	1.222806
std	1.168809	0.762923	0.521942	0.124262	0.888921
min	0.252250	0.212639	0.216400	0.228653	0.212639
25%	0.522855	0.320929	1.041617	0.566091	0.535564
50%	2.284141	0.619976	1.279806	0.587114	0.858973
75%	3.073016	1.642946	1.599099	0.679181	1.759015
max	4.215517	2.727565	2.269734	1.021070	4.215517

### Blind system validation

**Fig 7:**
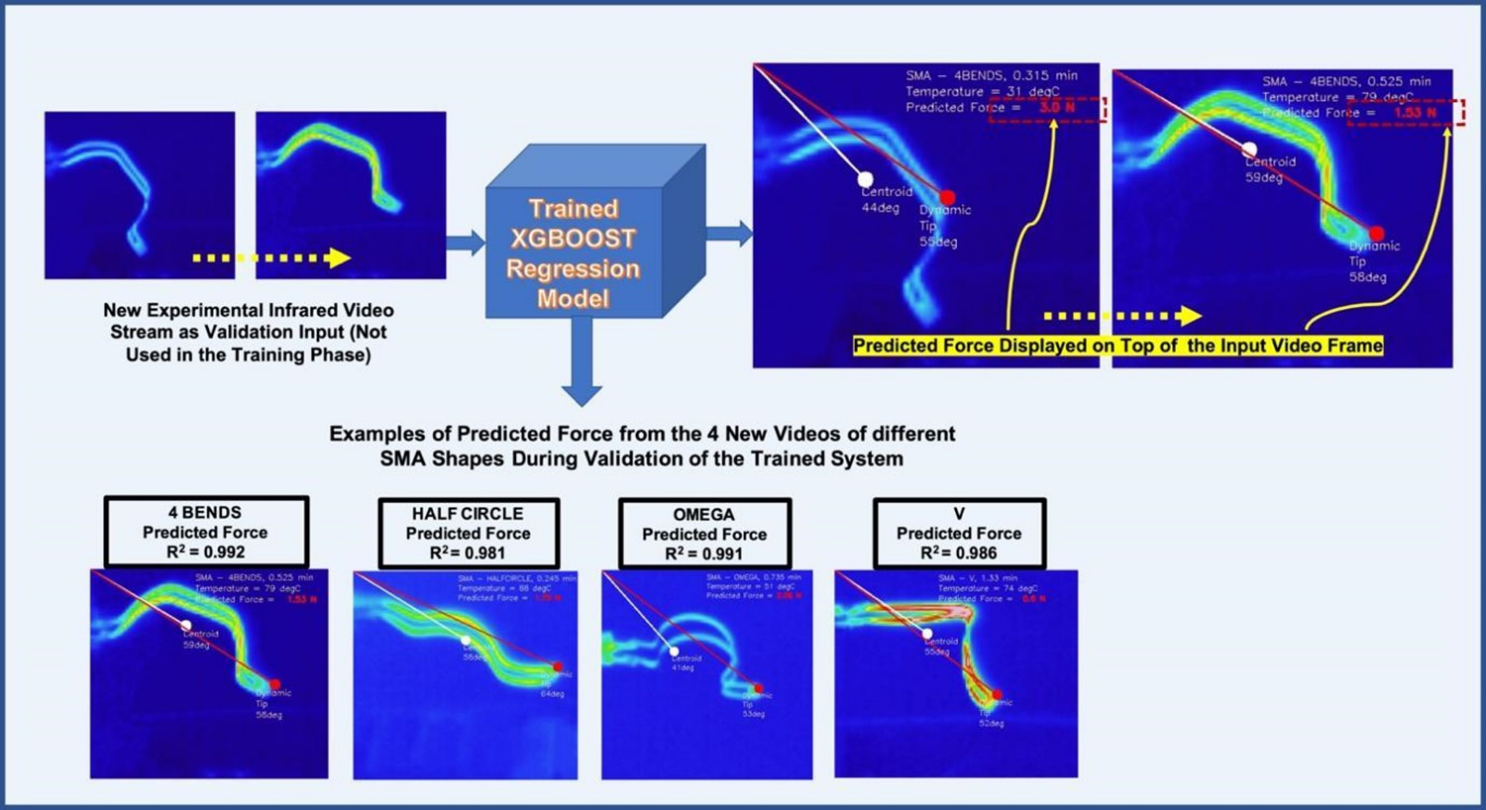
Results of prediction from the trained XGBoost model during the blind validation stage, where R^2^ score of predictions varied on a range of 0.981–0.992 and predicted force was displayed on top of the video frames dynamically.

The methodology consists of two test rigs with automated data logger, an infrared camera, a Computer Vision system for dynamically analysing video stream, coupled with the best trained XGBoost regression model was adapted as a predictive system (as shown in the Figs 4–6) to predict potential force from the video data collected using unknown SMA foil samples. The measured force from these samples were not used in the training and development of the model, rather kept separately for the blind validation. The overall predictive system was blind tested and validated to establish a performance accuracy and ultimate deploy ability of such a system. Results from the system blind validation are demonstrated in Fig 7, where R^2^ score of predictions varied on a range of 0.981–0.992 and predicted force was displayed on top of the video frames dynamically. System predicted force values were dynamically displayed on top of the video frames, with very few scenarios noisy predictive outcomes, due to presence of some insignificant and noisy video frames which were not eliminated from the validation video data, particularly during the transition between two consecutive excitement cycles.

### Comparative model selection

Five different regression models including AdaBoost Regressor, Extra Tree Regressor, Gradient Boosting Regressor, XGBoost Regressor, and Random Forest Regressor were employed to conduct a comparative analysis. Table 4(A)–4(E) summarises all results from the comparative predictive regression analysis, covering results from the four differently shaped SMA foils and an additional scenario while combining all shapes together. As evident from the tables, we found that XGBoost Regressor outperformed the other four ensemble regression models in both predicting aim A and aim B.

## Discussion

We aimed to achieve a complete solution as a system, novelty in each of the steps, including SMA material fabrication, digital material footprint generation, digital characterisation of a novel material, digital optimisation of material selection, and finally a working system that fully automates the whole process.

Here, we reported the new manufacturing process to achieve NiTi SMA foil of 30 mm width. This is a significant improvement in the field of SMA foils, bringing a major flexibility in designing the shape and the size of foils. This type of SMA foils were successfully produced by a planar flow casting facility at CSIRO, Australia. The fabrication of SMA foils is low-cost and can be produced in large volumes. This 30 mm SMA foil presents a unique possibility for a wide range of applications.

The fatigue resistance as well as other mechanical properties that are important for most of the industrial applications, except of limited space applications. Obtaining these properties are the essential part of our current efforts in investing the dependence of the microstructures and properties on processing. For this modelling manuscript, more consideration was given on the modelling role for mitigating the fatigue in the future development. One of futural extensions of the predictive modelling framework is the developing of its ability to anticipate the degradation of the SMA actuation as a result of materials fatigue and being able to take corrective controls by increasing the stimulus level in advance.

The optimisation of the microstructures through the process optimisation of the planar flow casting has been included in this study on the relationships of process-microstructure-actuation behaviour and other properties. It is a critical element that helped and validated the overall understanding of SMA behaviours. The developed AI based model will be further expanded to accommodate the effects of the varying microstructures on the actuation behaviour.

In the next phase, we used computer vision and XGBoost machine learning based Artificial Intelligence techniques, to develop a new methodology, which can rapidly and highly accurately predict key attributes, namely, generated force and actuation angle, to characterise many newly manufactured NiTi SMA foil. In summary, 95% overall accuracy was achieved when the system tried to predict possible actuation angle based on SMA foil body temperature. We also showed that 99.5% overall accuracy was achieved by the best model while predicting the possible amounts of force that could be generated by an actuating foil.

In combination with manufacturing facilities, digital imaging system, and Artificial Intelligence techniques, we developed a fully automated stand-alone system that is uniquely capable to characterize SMA materials, significantly improving material selection and material adaptability for a real-life application. Testing and blind validating the generalisation capability of the proposed system was an important step to be able to accept and trust such a system using Artificial Intelligence. Results from the blind validation stage indicated the validation accuracy of 98%-99%. This shows an opportunity to adapt predictive technologies for digital manufacturing in the era of Industry 4.0.
